# Improved A-Line and B-Line Detection in Lung Ultrasound Using Deep Learning with Boundary-Aware Dice Loss

**DOI:** 10.3390/bioengineering12030311

**Published:** 2025-03-18

**Authors:** Soolmaz Abbasi, Assefa Seyoum Wahd, Shrimanti Ghosh, Maha Ezzelarab, Mahesh Panicker, Yale Tung Chen, Jacob L. Jaremko, Abhilash Hareendranathan

**Affiliations:** 1 Department of Radiology and Diagnostic Imaging, University of Alberta, Edmonton, AB T6G 2R3, Canada; 2 Infocomm Technology Cluster, Singapore Institute of Technology, Singapore 828608, Singapore; 3 Department of Internal Medicine, Hospital Universitario La Paz, Paseo Castellana 241, 28046 Madrid, Spain

**Keywords:** lung ultrasound, TransUNet, boundary-aware dice loss, deep learning, medical image processing

## Abstract

Lung ultrasound (LUS) is a non-invasive bedside imaging technique for diagnosing pulmonary conditions, especially in critical care settings. A-lines and B-lines are important features in LUS images that help to assess lung health and identify changes in lung tissue. However, accurately detecting and segmenting these lines remains challenging, due to their subtle blurred boundaries. To address this, we propose TransBound-UNet, a novel segmentation model that integrates a transformer-based encoder with boundary-aware Dice loss to enhance medical image segmentation. This loss function incorporates boundary-specific penalties into a hybrid Dice-BCE formulation, allowing for more accurate segmentation of critical structures. The proposed framework was tested on a dataset of 4599 LUS images. The model achieved a Dice Score of 0.80, outperforming state-of-the-art segmentation networks. Additionally, it demonstrated superior performance in Specificity (0.97) and Precision (0.85), with a significantly reduced Hausdorff Distance of 15.13, indicating improved boundary delineation and overall segmentation quality. Post-processing techniques were applied to automatically detect and count A-lines and B-lines, demonstrating the potential of the segmented outputs in diagnostic workflows. This framework provides an efficient solution for automated LUS interpretation, with improved boundary precision.

## 1. Introduction

During the COVID-19 pandemic, healthcare systems faced immense challenges, often operating at full capacity or beyond. One of the key issues was the absence of effective triage methods to quickly identify patients in critical need of care. As COVID-19 primarily affects the respiratory system, lung involvement was recognized as a reliable indicator for assessing disease severity. This measure became essential not only for determining which patients required immediate intervention, but also for monitoring their progress over time. Assessing lung involvement has since proven valuable as a biomarker for tracking recovery and identifying complications, making it a crucial tool for evaluating lung health in both acute and long-term care.

In the current clinical care pathway, access to diagnostic ultrasound is often limited to a few tertiary hospitals that have well-trained sonographers and radiologists to interpret LUS images. Currently, lung examinations depend on expensive imaging technologies like X-ray, CT, and MRI, which are often inaccessible to remote communities and underserved populations, and may not be suitable for ongoing or periodic monitoring. These models fail to reach rural and remote centers where there is a need for continuous assessment of pulmonary health using low-cost imaging equipment. Point-of-care ultrasound (POCUS) [1] has emerged as a valuable diagnostic tool in these settings, due to its portability, safety, and affordability, offering a non-invasive, radiation-free, real-time, and cost-effective imaging solution. Unlike traditional imaging modalities, POCUS devices are compact and can be operated with minimal setup, making them ideal for bedside assessments. Additionally, their usability by lightly trained personnel, such as nurses, enhances their accessibility in various clinical settings. Despite these benefits, interpreting ultrasound images remains a significant challenge, as it requires specialized training. In POCUS images, identifying features such as A-lines and B-lines manually can be time-consuming. Hence, there is a need for automated tools to assist in image interpretation and ensure consistent diagnostic accuracy in LUS.

**Figure 1:**
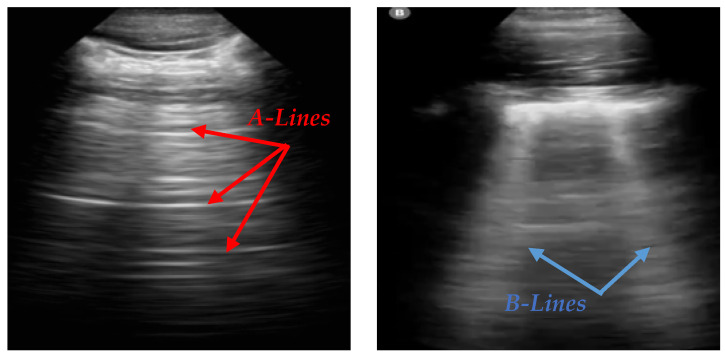
An illustration of A-lines and B-lines in an LUS image. A-lines are horizontal reverberation artifacts indicating normal aerated lung tissue, while B-lines are vertical, hyperechoic artifacts extending from the pleural line to the edge of the image, signifying potential lung abnormalities [5].

Lung POCUS shows different ultrasound patterns in COVID-19 compared to pulmonary edema, bacterial pneumonia, and other viral pneumonias, and is thus useful in differentiating between these conditions [2]. In LUS, A-lines and B-lines serve as crucial markers in the evaluation of lung health. A-lines are horizontal, reverberation artifacts seen in healthy, aerated lung tissue. These lines are typically observed when there is no abnormal fluid accumulation in the lungs, indicating normal lung conditions. B-lines are vertical, hyperechoic artifacts that extend from the pleural line to the bottom of the ultrasound image, often seen in pathological conditions. The presence of B-lines can indicate interstitial lung disease, pulmonary edema, or lung infections such as COVID-19 [3]. Various tools, like ExoLungAI, have been developed to detect these artifacts in ultrasound videos, categorizing them based on the quantity of A-lines and B-lines. Videos with more than five B-lines are classified as B-line cases, while those with dominant A-lines fall into a separate category [4]. Figure 1 illustrates the appearance of A-lines and B-lines in the lung ultrasound images used in this study.

Increasing numbers of B-lines can signify lung pathologies such as interstitial lung disease or pulmonary edema, or infection, particularly COVID-19. Accurate detection and quantification of these lines is essential for diagnosing and monitoring diseases by LUS, particularly in settings such as the COVID-19 pandemic. However, identifying A-lines and B-lines in ultrasound images poses several challenges. The quality of the ultrasound image can vary significantly depending on operator skill, patient anatomy, and equipment settings. Additionally, distinguishing these features requires expertise and a trained eye, which are often not available outside of tertiary hospital settings. This challenge is further compounded by the need to quickly and consistently analyze large numbers of images in high-demand clinical environments.

To address the challenges in interpreting lung ultrasound images, particularly in identifying A-lines and B-lines, we propose TransBound-UNet, a segmentation pipeline inspired by TransUNet [6], incorporating a transformer-based encoder and boundary-aware Dice loss. We use pre-processing steps like noise reduction and contrast enhancement to improve the visibility of key features. The model employs a hybrid architecture that integrates the strengths of transformers and convolutional neural networks (CNNs) for efficient segmentation. Additionally, we propose a composite loss function that combines Dice loss for overall segmentation optimization with a boundary-aware component, ensuring the separation of adjacent regions and reducing the likelihood of unintended connections. Post-processing is then applied to analyze the segmented regions and accurately detect and count A-lines and B-lines, offering a solution for LUS analysis.

The main contributions of this work are as follows:

- Proposing a lightweight model, inspired by the architecture of TransUNet, that combines transformer components with a convolutional network. We hypothesize that this reduction in architectural complexity improves the generalizability of the model.
- Designing a novel loss function that integrates classic Dice loss with a boundary-weighted penalty, improving segmentation performance, particularly around the boundaries of target regions.
- Developing a post-processing pipeline for counting A-lines and B-lines, enabling efficient and accurate analysis of lung ultrasound images.

The rest of this paper is organized as follows: Section 2 describes the related work, summarizing previous efforts in lung ultrasound analysis, including methods for A-line and B-line detection. Section 3 presents our proposed pipeline. Section 4 outlines the experimental setup, including data sources and implementation details. Section 5 provides the discussion. Section 6 concludes the paper and outlines future work.

## 2. Related Work

The analysis of LUS images, particularly the detection of A-lines and B-lines, has been a growing research area, due to the importance of these features in diagnosing and tracking respiratory disorders. This section reviews the existing literature relevant to our work, categorized into three main areas: A-line and B-line segmentation, automated analysis of LUS, and medical image segmentation techniques.

### 2.1. A-Line and B-Line Segmentation

Early approaches for automatic segmentation of A-lines and B-lines in lung ultrasound relied on classical image processing techniques. Algorithms such as edge detectors, along with the Hough Transform, were used for edge line detection [7]. Traditional machine learning techniques complemented these methods: PCA and Independent Component Analysis (ICA) facilitated feature extraction, while classifiers like SVM, Random Forest, KNN, and logistic regression were applied for classification tasks. SVM, in particular, was preferred in COVID-19 LUS analyses for its robustness with high-dimensional data [8]. Despite their effectiveness in certain datasets, these approaches often gave poor results when validated on images of variable quality, complex anatomical structures, and overlapping artifacts, hence limiting their broader clinical application.

Deep learning methods were widely utilized for LUS segmentation and classification. Convolutional neural networks, including U-Net and its variants, were successfully used for identifying and segmenting A-lines and B-lines by learning hierarchical features directly from data. For instance, Howell et al. [9] developed a lightweight U-Net for multi-class segmentation of ribs, pleural lines, and artifacts, demonstrating promising results, even with limited clinical data. Advanced architectures further expanded the scope of DL in LUS. Shea et al. [10] combined LSTM-CNN frameworks for video-level pleural effusion detection and frame-level B-line annotation. Ebadi et al. [11] utilized the Kinetics-I3D network for rapid and reliable interpretation of ultrasound videos. For addressing challenges like feature blurring, Yuanlu Ni et al. [12] proposed MEVAL (Multi-Enhanced Views Active Learning), a method that improved classification accuracy for patterns such as A-lines, B-lines, consolidation, and pleural effusion. Nekoui et al. [4] evaluated the performance of ExoLungAI, a tool designed to visualize A-lines and B-lines in lung ultrasound videos. The tool counts B-lines based on the affected rib space, and classifies videos by comparing the number of affected frames to a threshold. Videos with more than five B-lines are categorized as B-line cases, while those with significant A-line presence are classified accordingly. An improved Faster R-CNN model was developed to accurately locate the pleural line, followed by segmentation of the LUS image below the pleural line to exclude interference from similar structures [13]. Image processing techniques, including total variation, matched filter, and gray difference, were then applied for automatic A-line detection.

### 2.2. Automated Analysis of Lung Images

The integration of AI into lung imaging has revolutionized diagnostic approaches, particularly for lung ultrasound. AI-based techniques aim to enhance accuracy, reduce variability, and enable automated analysis, addressing challenges in traditional interpretation methods.

A review highlighted AI’s transformative role in LUS, especially for COVID-19 pneumonia [8]. LUS combined with AI is a safe and cost-effective tool that improves diagnostic accuracy and reduces variability. It also supports remote diagnostics through innovations like automated scoring and telerobotic systems. However, challenges like poor image quality, operator dependency, and limited datasets hinder model generalizability. Despite this, AI-integrated LUS and telehealth technologies promise real-time monitoring and broader healthcare advancements.

Another review [14] on deep learning applications in LUS for COVID-19 provided a comprehensive analysis of ultrasound systems, datasets, and various deep learning models, including CNNs, U-Net [15], ResNet [16], DenseNet [17], Inception [18], Mo-bileNet [19], NASNet [20], and COVID-CAPS [21]. The authors concluded that these models show significant potential in aiding COVID-19 diagnosis through LUS. They review also highlighted several challenges in developing deep learning models for LUS analysis, including operator dependency, the need for diverse datasets, and limitations in model interpretability. Addressing these challenges is crucial for improving model reliability in clinical settings.

A 2024 paper [22] explores key aspects of Computer-Aided Diagnosis Systems (CADSs) leveraging deep learning methods for diagnosing COVID-19. It covers segmentation, classification, explainable AI (XAI), and predictive research across various medical imaging modalities, including CT, ultrasound, and X-rays. The study highlights models such as CNNs, autoencoders [23], GANs [24], attention mechanisms [25], transformers [26], and RNNs [27]. In the segmentation section, architectures like SegNet [28] and U-Net are discussed. The authors also enumerate challenges, including distribution shifts, generalization issues, and resource limitations, emphasizing the need for robust and adaptable solutions in CADS development.

### 2.3. Medical Image Segmentation Techniques

Deep learning has significantly advanced medical image analysis, with improvements in both classification and segmentation. Hussain et al. [29] enhanced explainability in classification tasks by integrating pretrained VGG-19 with Grad-CAM, improving diagnostic support through better visualization. Similarly, Alam et al. [30] focused on fracture prediction using X-ray image analysis, incorporating YOLOv8 for ROI detection and evaluating multiple classification algorithms.

Parallel to these advancements in classification, medical image segmentation has rapidly evolved with the development of deep convolutional neural networks (CNNs), transformers, and hybrid architectures. Among these, TransAttUnet [31] stands out as a notable Transformer-based Attention Guided Network designed to enhance semantic segmentation. By integrating multi-level guided attention and multi-scale skip connections, TransAttUnet strengthens feature representation across different resolutions.

Recent approaches in medical image segmentation have explored the integration of attention mechanisms, graph neural networks [32], and domain adaptation techniques [33] to further improve performance. Methods like nnU-Net [34] have set benchmarks by automating hyperparameter selection and achieving exceptional generalizability across various medical imaging modalities. Hussain et al. [35] proposed MAGRes-Unet, which enhances U-Net with multi-attention gate modules and residual blocks to improve feature learning and focus on small-scale tumors.

Transformer-based architectures, including TransUNet [6], have demonstrated the potential to bridge local and global feature learning, making them highly effective for tasks requiring both fine-grained and holistic contextual understanding. The Swin Transformer [36] is a hierarchical vision transformer that partitions the image into non-overlapping windows and shifts them between layers to capture both local and global dependencies efficiently.

TransUNet is a hybrid architecture combining the strengths of U-Net and Transformers, and has gained significant attention in medical image segmentation. By embedding Transformer layers into the encoder, TransUNet leverages self-attention mechanisms to capture long-range dependencies, while maintaining the localization capabilities of the U-Net’s convolutional structure. This hybrid design makes TransUNet particularly well suited for tasks where both local boundary precision and global context are critical. However, vanilla TransUNet has shown uneven performance in ultrasound segmentation, due to the highly variable nature of ultrasound images, which contain speckle noise, low contrast, and heterogeneous anatomical structures. These challenges necessitate a more robust and adaptive approach that integrates transformer components with a lightweight convolutional network to enhance both segmentation consistency and efficiency.

## 3. Methodology

**Figure 2:**
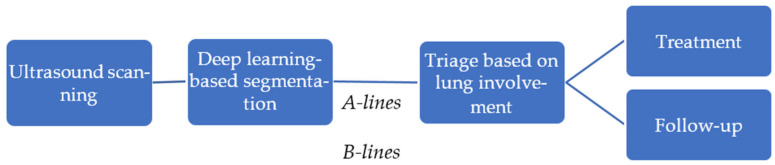
An overview of the automated pipeline for lung ultrasound analysis.

In this study, we propose an automated pipeline for lung ultrasound image segmentation to identify clinically relevant features, such as A-lines and B-lines, aimed at assisting in the management of COVID-19 patients. Ultrasound scanning plays a pivotal role in bedside evaluation, especially in emergency triage settings, offering a rapid, non-invasive assessment of lung involvement. Using deep learning-based segmentation techniques, clinically relevant features, such as A-lines and B-lines, are identified, providing critical insights into lung pathology. This automated analysis enables efficient triage by evaluating the extent of lung involvement, guiding immediate treatment decisions, and facilitating long-term monitoring of patients, especially in the case of Long COVID. Figure 2 illustrates the steps involved in the pipeline. It shows how segmentation results can be integrated into clinical workflows to support personalized treatment plans and follow-up strategies, ultimately enhancing patient care and resource management in healthcare settings.

To achieve accurate and efficient lung ultrasound A-line and B-line counting and segmentation, the proposed methodology uses pre-processing, deep learning-based segmentation, and post-processing. These stages help to improve image quality and make it easier to extract important features. They also provide clear outputs that support better clinical decision-making. The proposed approach, TransBound-Unet, incorporates a transformer-based encoder to capture long-range dependencies, a Unet-style decoder, and a boundary-aware Dice loss to refine segmentation along object boundaries.

### 3.1. Pre-Processing for Ultrasound Image Segmentation

Pre-processing enhances ultrasound image quality, reduces noise, and standardizes input to improve training efficiency and model performance. In this study, non-local means filtering is applied to reduce noise and mitigate artifacts that are commonly found in ultrasound imaging. Contrast-Limited Adaptive Histogram Equalization (CLAHE) enhances the visibility of subtle features, such as A-lines and B-lines, that are crucial for clinical interpretation. Additionally, unsharp masking improves edge clarity, aiding in the identification of feature boundaries.

While these steps enhance data quality, the pre-processing pipeline is intentionally kept minimal to preserve critical features and leverage the deep learning model’s ability to learn directly from raw data. This balanced approach ensures that the model remains robust to diverse input conditions, while benefiting from improved image quality.

### 3.2. Deep Learning Architecture for A-Line and B-Line Segmentation

Inspired by TransUNet, we propose an improved segmentation framework tailored for lung ultrasound analysis. While transformer-based architectures have demonstrated strong performance in medical imaging, they typically require large amounts of data for optimal results. However, in tasks with limited or less diverse datasets, these models can be more sensitive to training variations and dataset characteristics. This variability is influenced by factors such as the dataset’s distribution, the composition of the training, validation, and test splits, and the stochastic nature of the training process. These effects are particularly noticeable in datasets with unique or non-uniform distributions, where model generalization can be more challenging.

**Figure 3:**
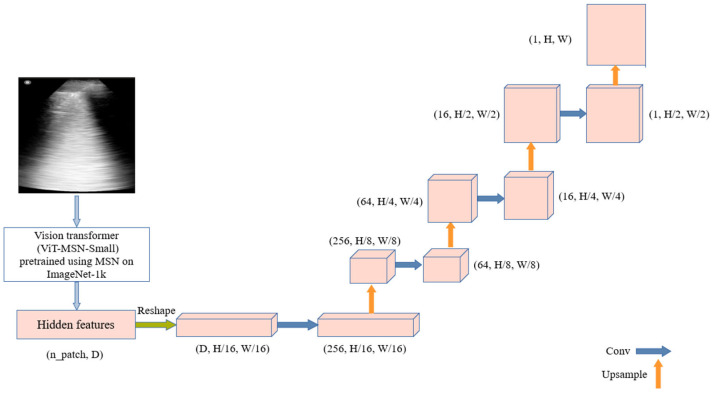
TransUNet-Inspired architecture: combining vision transformer features with upsampling and convolution for ultrasound segmentation.

The process begins with ViT-MSN-Small, a vision transformer pretrained using Masked Siamese Networks (MSN) on ImageNet-1k, serving as a powerful backbone for feature extraction. The output of the ViT consists of hidden features, initially represented in a flat, patch-based form. These hidden features are reshaped into a spatial tensor, representing a coarse resolution of the original image. The decoder utilizes this low-dimensional tensor to reconstruct the segmentation mask. It is composed of multiple upsampling layers followed by convolutional layers, progressively refining the segmentation output. The architecture is illustrated in Figure 3.

- Loss Function

In the segmentation of A-lines and B-lines in lung ultrasound images, accurately preserving the boundaries between these lines and surrounding tissues is crucial for reliable diagnostic analysis. The close proximity of A-lines and B-lines, as well as their potential intersections with other structures such as lung tissue or artifacts, presents a significant challenge for segmentation. In LUS images, the tissue textures are complex, and the boundaries between A-lines, B-lines, and surrounding tissues are often blurred. We propose a boundary-aware loss function to improve segmentation performance.

To the best of our knowledge, while boundary-aware loss functions have been explored in general segmentation tasks, their application to A-line and B-line segmentation in lung ultrasound images remains relatively under-explored. In traditional segmentation tasks, boundary-aware losses have been successfully employed to enhance segmentation accuracy near object edges, allowing the model to preserve fine details, even in regions with closely spaced structures [37]. In medical image segmentation, Li [38] proposed a geometric boundary constraint loss that utilizes the boundary information of the target object. This information applies geometric constraints on the segmentation process, giving higher weights to the boundary areas for more accurate segmentation.

The proposed loss function combines the classic Dice loss with a boundary-weighted penalty to improve segmentation performance, especially around the boundaries of target regions. The Dice loss is used to measure the overlap between the predicted segmentation mask and the ground truth mask. It is defined as follows:

(1) $$Dice\;Loss=1-\frac{2.\left|Intersection(pred,\;target)\right|+\epsilon }{\left|pred\right|+\left|target\right|+\epsilon }$$

Here, ϵ is a small constant added to avoid zero division when both the target and the prediction are empty masks. This loss encourages maximizing the overlap between the prediction and target, while minimizing false positives and false negatives.

To enhance segmentation accuracy, particularly near region boundaries, the Boundary Loss applies a weighted penalty to areas close to the ground truth mask boundaries. The boundary of the ground truth mask is a binary mask that highlights pixels along the inner boundary of the target regions. This boundary mask is used to weight the Binary Cross Entropy (BCE) loss, emphasizing errors near the boundaries and ensuring that the model pays more attention to accurately segmenting these critical areas. The Boundary Loss is defined as follows:

(2) $$Boundary\;Loss=BCE\left(pred,\;target\right)\;.\;boundary\;mask$$

The final Boundary-Aware Dice loss combines the Dice loss and the weighted Boundary Loss as follows:

(3) Boundary Aware Dice Loss=Dice loss+λ . Boundary loss

### 3.3. Post-Processing for A-Line and B-Line Detection and Counting

After segmentation, various image processing operations were used to detect and classify lines in the segmented image as A-lines vs. B-lines. The segmented output was converted to a binary format and dilated. Contours were extracted and classified based on their geometric properties. Algorithm 1 shows a short pseudocode of the post-processing steps.
**Algorithm 1**. Pseudocode for post-processing pipeline to detect and count A-lines and B-lines in lung ultrasound images***Input:**** Segmented image* *Convert the image to binary format and dilate using a small kernel.* *Apply Gaussian blur and Canny edge detection.* *Dilate the edges for better contour detection.* *Extract contours from the processed image.* ***For**** contour in contours:*
  *Calculate the bounding box dimensions (width, height).*
  *Classify as A-line if width > 1.5 × height.*
  *Classify as B-line if height > 1.5 × width.*
***If***
* no contours are detected, retain the original segmented image.*
***Output***
*: Count of A-lines and B-lines, visualization.*


## 4. Experiments and Results

The dataset used in this study consists of 2D LUS images obtained from subjects from Hospital Universitario Puerta de Hierro, Majadahonda, Spain [39]. Ultrasound exams were performed with the patient in supine or sitting positions. The probe was placed obliquely along anatomical lines at the 2nd–4th ICS (parasternal, midclavicular, anterior axillary, midaxillary) and 2nd–10th ICS (paravertebral, sub-scapular, posterior axillary). The dataset includes a total of 4599 images, each accompanied by corresponding segmentation labels, without pre-determination or counting of the presence of A-lines and B-lines in each image.

For model development, 5-fold cross-validation was applied. Experiments were conducted on a Lambda laptop, manufactured by Clevo Co. (New Taipei City, Taiwan), with an NVIDIA RTX 3080 GPU, using PyTorch 2.5.1 for implementation. Before training, images were normalized using the pre-processing steps described in Section 3.1. The model was trained using the Adam optimizer with a learning rate of 1 × 10^−4^, a batch size of 16, and a weighted sum of Dice and boundary-aware loss to enhance segmentation. Training was conducted for 40 epochs.

To evaluate the performance of the proposed models, we utilized a diverse range of metrics.

The Dice Score measures the overlap between the predicted segmentation and the ground truth. The formula is given as follows:


(4) $$Dice=\frac{2\times \left|predicted\;segmentation\cap ground\;truth\right|}{\left|predicted\;segmentation\right|+\left|ground\;truth\right|}$$

Sensitivity, also referred to as Recall or the true positive rate, measures the ability to correctly detect true positives. Specificity, or the true negative rate, assesses the model’s ability to accurately identify true negative classes, such as the background class. These can be identified using the following formulas:


(5) $$\mathrm{Sensitivity}=\frac{TP}{TP+FN}$$

(6) $$\mathrm{Specificity}=\frac{TN}{TN+FP}$$

TP (true positive) denotes the pixels correctly predicted as belonging to the target class. FP (false positive) represents the pixels incorrectly classified as the target class.

FN (false negative) indicates the pixels that belong to the target class but are incorrectly classified as the background. TN (true negative) signifies the pixels correctly predicted as belonging to the background or non-target class.

Hausdorff Distance (HD) is a metric used to evaluate how well the predicted boundary matches the ground truth boundary. HD is mathematically expressed as follows:


(7) $$\mathrm{HD}(A,\;B)=\left(\underset{a\in A}{\max}\underset{b\in B}{\min}\left\|a-b\right\|,\;\;\underset{b\in B}{\max}\underset{a\in A}{\min}\left\|a-b\right\|\right)$$

Let *A*, *B* be two sets of points representing the boundaries of the predicted segmentation and ground truth. Let *a* and *b* represent individual points from sets A and B, respectively. The term $\left\|a-b\right\|$ is the Euclidean distance between the two points. For each point in the set, the closest point in set *B* is identified, and vice versa. The Hausdorff Distance is then calculated by taking the maximum of these minimum distances. This ensures that the HD captures the worst-case discrepancy, meaning the largest mismatch between the two sets.

Precision is another metric used to measure the accuracy of positive predictions made by a model. The formula is as follows:


(8) $$\mathrm{Precision}=\frac{TP}{TP+FP}$$

The F1 Score is the harmonic mean of Precision and Recall. Maximizing the F1 Score ensures a balance between Precision and Recall, improving both simultaneously. It can be formulated as follows:


(9) $$F1\;=2\times \frac{Precision\times Recall}{Precision+Recall}$$

Finally, Intersection over Union (IoU) is a widely used metric for evaluating the accuracy of segmentation models. It measures the overlap between the predicted segmentation mask and the ground truth mask. The formula for IoU is as follows:


(10) $$IoU=\frac{\left|predicted\;segmentation\cap ground\;truth\right|}{\left|predicted\;segmentation\cup ground\;truth\right|}$$

In our experiments with this dataset, TransUNet achieved high Dice Scores, reaching up to 88%. However, its performance varied across runs, with Dice Scores ranging from 0.64 to 0.88. Over 10 runs, the model achieved an average Dice Score of 75%. This variability highlights the challenges of segmentation consistency, particularly in datasets with diverse imaging conditions. To address this, we propose a method that integrates transformer components with a lightweight convolutional network, aiming to enhance both robustness and efficiency.

Table 1 presents a comparison of the TransBound-Unet, with and without the boundary-aware loss, against several state-of-the-art networks for medical image segmentation. These include U-Net [15], a widely used encoder–decoder architecture for biomedical segmentation. Attention U-Net [40] incorporates attention mechanisms to enhance focus on relevant regions. UNETR [41] is a transformer-based model that leverages long-range dependencies in volumetric data. SwinUNETR [42] combines the hierarchical features of Swin Transformers with UNETR to enhance both global and local feature extraction. TransUNet [6] integrates transformers and CNNs to balance global context with local spatial details. Additionally, we include a recent lightweight U-Net variant [9], specifically designed for LUS segmentation. This evaluation demonstrates the effectiveness of the proposed method in lung ultrasound segmentation. The results show that the proposed method with the boundary-aware loss consistently outperforms the other approaches across most metrics, demonstrating its superior performance in segmenting lung ultrasound images. Specifically, it achieves the highest Dice Score, indicating the best overlap between predicted and ground truth segmentation.

**Figure 4:**
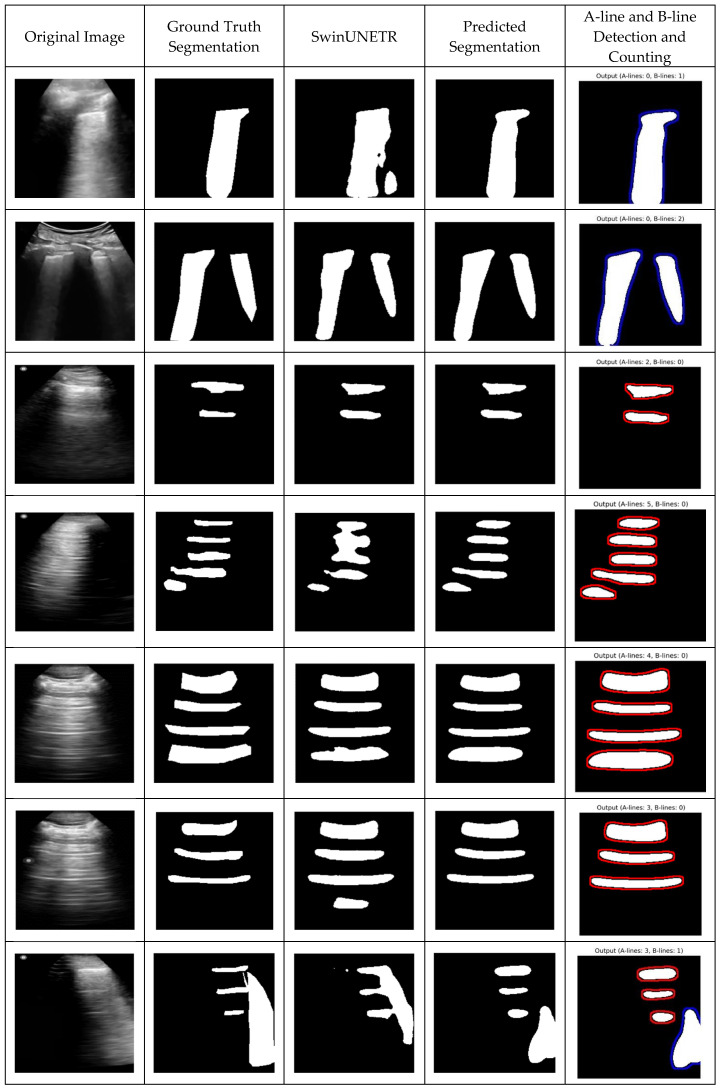
Qualitative results—predicted segmentations with A-line and B-line detection and counting. Each row presents different cases: (1) original lung ultrasound image, (2) ground truth segmentation, (3) SwinUNETR-predicted segmentation, (4) TransBound-UNet-predicted segmentation, and (5) A-line and B-line detection and counting. The identified artifacts, such as A-lines (horizontal reverberation) and B-lines (vertical hyperechoic artifacts), are highlighted in the final column.

Following the segmentation performance analysis, the qualification results and post-processing steps for identifying and counting A-lines and B-lines were evaluated. After obtaining the segmentation masks, a post-processing pipeline was applied to detect lines indicative of A-lines and B-lines. The results demonstrate the proposed method’s high precision in distinguishing between these artifacts (Figure 4). Additionally, SwinUNETR achieved the second-best segmentation performance, and its results are also presented in Figure 4 for comparison. While the performance of other approaches was relatively close, SwinUNETR outperformed Attention U-Net by a small margin.

## 5. Discussion

This study introduces a novel deep learning-based framework, TransBound-UNet, for the detection and segmentation of A-lines and B-lines in LUS images. This task is crucial for the diagnosis of pulmonary conditions, where accurate segmentation of these lines can provide vital insights into the presence of diseases like pulmonary edema or interstitial lung disease. By simplifying the TransUNet architecture and incorporating a Boundary-Aware Dice loss into a hybrid Dice-BCE loss function, TransBound-UNet achieved significant improvements in segmentation accuracy, particularly along the boundaries of A-line and B-lines.

TransUNet has demonstrated significant potential in medical image segmentation by effectively combining convolutional layers with transformer blocks, capturing both local and global dependencies. Inspired by the success of TransUNet, we proposed a simplified architecture that leverages the strengths of transformers while reducing architectural complexity. TransBound-UNet maintains the core concept of using a Vision Transformer (ViT) for global feature extraction, specifically employing ViT-MSN-Small, a model pretrained using MSN on ImageNet-1k. To enhance efficiency and adaptability for binary classification tasks, such as lung ultrasound segmentation, we introduced a lightweight decoder.

TransBound-UNet and TransUNet both use a ViT backbone and a UNet-like decoder. However, TransBound-UNet differs by removing skip connections and simplifying the decoder to a lightweight convolutional structure. Instead of using both convolutional and transformer features, it relies entirely on transformer-derived features. These changes make the architecture more efficient by significantly reducing computational cost. Compared to the original TransUNet, TransBound-UNet lowers the FLOPs from 27.06 GFLOPs to 4.57 GFLOPs, an 83% reduction. The number of trainable parameters also decreases from 63 M to 23 M, reducing memory usage and making the model more lightweight and practical.

The proposed loss function enhances segmentation performance, particularly in capturing fine boundary details. By incorporating boundary-specific penalties, it improves edge delineation, which is crucial in medical imaging. The function balances global and local performance by combining Dice loss for overall overlap optimization with a boundary-aware component for precise edge segmentation. Its adaptability, through an adjustable weight parameter, allows for customization based on the specific characteristics of different datasets and tasks. Inspired by the need to address complex boundaries in medical imaging, this loss function ensures reliable and precise segmentation, supporting effective diagnosis and treatment planning.

Our developed model demonstrated excellent performance across multiple key metrics, often outperforming or matching other state-of-the-art methods. Its Dice Score was the highest among the compared models, highlighting the model’s strong capability in segmenting accurately. The model also excelled in Specificity, indicating its strong ability to exclude irrelevant regions from the segmentation mask, which is important for reducing false positives. Additionally, its Hausdorff Distance, a key measure of boundary accuracy, was lower than that of all the other models, by a considerable margin, demonstrating that TransBound-UNet produces more precise segmentation boundaries. The overall performance, reflected by the F1 Score and IoU, further highlights the model’s robustness.

Beyond segmentation, this approach could benefit downstream tasks such as disease detection and classification. For example, greater segmentation accuracy can enhance lesion detection and organ boundary delineation, ultimately improving diagnostic precision and clinical decision-making, particularly for lung diseases like COVID-19.

## 6. Conclusions and Future Works

In this work, we propose a lightweight deep learning architecture for lung ultrasound segmentation, leveraging the vision transformer ViT-MSN-Small. TransBound-UNet achieves high segmentation accuracy, while significantly reducing the number of trainable parameters compared to conventional deep learning models, making it a more computationally efficient solution for medical imaging applications. By incorporating a custom loss function designed to enhance boundary delineation, our model effectively captures fine anatomical structures, leading to a substantial reduction in the Hausdorff Distance. The results demonstrate that despite its reduced complexity, our method outperforms state-of-the-art architectures in key metrics such as Dice Score, Precision, and IoU, while maintaining high Specificity and Recall. This improvement highlights the potential of our approach for real-world deployment, particularly in resource-constrained clinical settings.

While TransBound-UNet shows promising results, several areas need further exploration. One major challenge in ultrasound imaging is its high operator dependence, which can lead to variability in multi-site datasets. Future research should focus on improving the model’s robustness to these inconsistencies, ensuring reliable performance across different users and imaging conditions.

Another important direction is incorporating self-supervised or semi-supervised learning to make better use of limited labeled data. Additionally, enhancing model interpretability would help clinicians to trust and understand the model’s decisions.

Finally, to assess its real-world impact, future studies should evaluate whether the model improves diagnostic accuracy—particularly, in clinical sensitivity and specificity for detecting lung diseases, including COVID-19—compared to state-of-the-art methods.

## Figures and Tables

**Table 1:** A performance comparison of TransBound-Unet (with and without Boundary Loss) against state-of-the-art networks. The best-performing results in each metric are highlighted in **bold**. The arrows in the table represent the desired direction for each metric: an upward arrow (↑) indicates that a higher value is preferable, while a downward arrow (↓) indicates that a lower value is desired.

Metric	Proposed (With Boundary Loss)	Proposed (No Boundary Loss)	U-Net [15]	Attention U-Net [40]	UNETR [41]	SwinUNETR [42]	TransUNet [6]	Lightweight UNET [9]
Dice Score ↑	**0.80**	0.79	0.746	0.752	0.75	0.753	0.75	0.745
Sensitivity ↑	0.83	0.84	0.83	**0.85**	0.79	0.84	0.81	0.83
Specificity ↑	**0.97**	0.96	0.95	0.95	**0.97**	0.95	0.96	0.95
Hausdorff Distance ↓	**15.13**	23.4	34.35	31.56	29.41	29.87	34.52	38.96
Precision ↑	**0.85**	0.82	0.77	0.77	0.82	0.78	0.81	0.75
F1 Score ↑	**0.84**	0.83	0.80	0.81	0.80	0.81	0.81	0.79
IoU ↑	**0.73**	0.71	0.67	0.68	0.67	0.68	0.67	0.65

## Data Availability

Data will be made available on request.
